# Chromosomal mosaicism goes global

**DOI:** 10.1186/1755-8166-1-26

**Published:** 2008-11-25

**Authors:** Ivan Y Iourov, Svetlana G Vorsanova, Yuri B Yurov

**Affiliations:** 1 National Research Center of Mental Health, Russian Academy of Medical Sciences, Moscow, 119152, Russia; 2 Institute of Pediatrics and Children Surgery, Rosmedtechnologii, Moscow, 127412, Russia

## Abstract

Intercellular differences of chromosomal content in the same individual are defined as chromosomal mosaicism (alias intercellular or somatic genomic variations or, in a number of publications, mosaic aneuploidy). It has long been suggested that this phenomenon poorly contributes both to intercellular (interindividual) diversity and to human disease. However, our views have recently become to change due to a series of communications demonstrated a higher incidence of chromosomal mosaicism in diseased individuals (major psychiatric disorders and autoimmune diseases) as well as depicted chromosomal mosaicism contribution to genetic diversity, the central nervous system development, and aging. The later has been produced by significant achievements in the field of molecular cytogenetics. Recently, *Molecular Cytogenetics *has published an article by Maj Hulten and colleagues that has provided evidences for chromosomal mosaicism to underlie formation of germline aneuploidy in human female gametes using trisomy 21 (Down syndrome) as a model. Since meiotic aneuploidy is suggested to be the leading genetic cause of human prenatal mortality and postnatal morbidity, these data together with previous findings define chromosomal mosaicism not as a casual finding during cytogenetic analyses but as a more significant biological phenomenon than previously recognized. Finally, the significance of chromosomal mosaicism can be drawn from the fact, that this phenomenon is involved in genetic diversity, normal and abnormal prenatal development, human diseases, aging, and meiotic aneuploidy, the intrinsic cause of which remains, as yet, unknown.

## 

Chromosomal mosaicism was originally defined as the presence of cells differing with respect to their chromosome complement in the same individual [1]. Although chromosomal mosaicism is repeatedly registered during cytogenetic analysis, one of the commonest genetic tests in medical genetics [2], its significance remains usually underappreciated. Nonetheless, during the last decade, a growing amount of studies has demonstrated that chromosomal mosaicism does contribute to human diversity [3-7], diseases [2,4,5,7-12], early prenatal brain development [3,13], and aging [14]. However, the real biomedical meaning of chromosomal mosaicism in humans is hardly known.

One of the previous studies published in *Molecular Cytogenetics *[15] has brought evidences that chromosomal mosaicism plays a role in the generation of meiotic aneuploidy known to be the leading genetic cause of human prenatal death and congenital malformations/learning disabilities [4,5,16]. Studying chromosome 21 in ovarian cells of normal female foetuses, Prof. Maj Hulten and her colleagues were able to give experimental support for their original hypothesis suggesting meiotic aneuploidy in human conceptuses to be the result of ovarian germline mosaicism that is produced during the normal prenatal development [15]. The data fit well with current concepts in biology of aneuploidy, essentially drawn from studies of trisomy 21 (Down's syndrome) [16]. More specifically, these findings have the potential to explain maternal age effect, recurrence of aneuploidy at subsequent conceptions, and abnormal maternal recombination patterns previously found *via *linkage analyses [15]. Although the idea put forward in this article has revolutionized our thinking about maternal meiotic aneuploidy suggesting mitotic aneuploidy to lie at the origin of meiotic aneuploidy, there was a strong experimental background for this hypothesis. Firstly, it has been recently noticed that chromosomal mosaicism is frequent among human foetuses, achieving the rate of 25% in spontaneous abortions [17]. Additionally, the confinement of chromosomal mosaicism to the specific tissue is a known phenomenon. As early as 1983, Kalousek and Dill have described the existence of chromosomal mosaicism exclusively confined to the placenta (confined placental mosaicism) [18]. About a year ago, there have been shown that somatic chromosomal mosaicism confines to the developing human brain in a significant proportion of normal human conceptions. Furthermore, it has been established that increase of mosaic aneuploidy in the developing human brain is an integral component of the human prenatal central nervous system development [13].

Therefore, one can conclude: (i) chromosomal mosaicism is extremely frequent in human foetuses; (ii) chromosomal mosaicism confines as to extraembryonic tissues (placenta) as to embryonic tissues (central nervous system and ovarian tissue). It is reasonable to suspect, that the later could be one of the major source for human tissue-specific pathology or multi-system diseases (including those that arise due to meiotic errors), as exemplified by M. Hulten and colleagues [15] as well as previous publications [4,5,7-12,17]. To understand whether chromosomal mosaicism has the potential to mediate intercellular diversity (somatic genome variations in unaffected individuals), one should address studies performed to reveal the real rate of cell-to-cell chromosomal number variability in unaffected human tissues [3-6,13-15,18-24] (Table 1). It is to note that almost all tissues, if thoroughly analyzed by a molecular cytogenetic technique, exhibit aneuploid cells. Thus, we can highlight the major difficulty for studies targeted at revealing effects of chromosomal mosaicism referred to the definition of non-pathogenic level of aneuploidy in a tissue. Therefore, an association between chromosomal mosaicism and an alteration to cellular/tissular physiology requires thorough control study of unaffected individuals (tissues).

**Table 1:** Chromosomal mosaicism in presumably normal human tissues.

Tissue	Description	References
Ovarian tissues	Small, but significant proportion of aneuploid cells (trisomy 21) in ovarian tissues of normal female fetuses	[15]
	
	15–20% of human oocytes	[19]

Sperm	2–10% of spermatozoa (0.1–0.2% per chromosome)	[20]

Chorionic villi	approaching 24% (~1% of aneuploid cells per chromosome)	[13]

Fetal human brain	approaching 30% (~1.5 of aneuploid cells per chromosome) 35% including chromosomal mosaicism confined to the fetal brain	[3,13]

Placenta	No generalized data; chromosomal mosaicism observed in ~2% of foetuses (9–11 weeks of gestation) referred to prenatal diagnosis	[21]

Skin (adults)	2,2% and 4,4% (in young and old individuals, respectively)	[22]

Liver (adults)	~3%	[23]

Blood (adults)	1–2% (randomly selected autosomes) and 3% (chromosome X)	[24]

Adult human brain	0.1–0.7% (autosomes and chromosome Y), 2% (chromosome X); tending to approach 10%, in total	[3,4,6]

Focusing on diseases associated with chromosomal mosaicism, one can note the broad spectrum of pathology associated with this type of somatic genomic variations from cases of chromosomal syndromes to complex neuropsychiatric and immune diseases [2-13,19]. Prof. Hulten and colleagues [15] have added meiotically originated aneuploidy syndromes to the "chromosomal mosaicism disease list". Furthermore, it suggests the commonest genetic cause of prenatal deaths to arise from chromosomal mosaicism, as well. Table 2 overviews current knowledge about chromosomal mosaicism contribution to human prenatal mortality and postnatal morbidity. We may conclude that the confinement of chromosomal mosaicism is likely to be the reason of tissue-specific dysfunction as exemplified by brain diseases and fetal brain and ovarian tissues [4,5,7,8,12,13,15]. Consequently, attempts to identify the role of chromosomal mosaicism in human pathology should directly evaluate malfunction tissue. Unfortunately, due to limited availability of the majority of human tissues for extended genetic studies and complexity of molecular cytogenetic analyses of low-level aneuploidy, such evaluations are rare. To date, only neural and ovarian tissues were assessed by high-resolution molecular cytogenetic techniques [3,6,8,12,13,15,25]. Nevertheless, tissues (cell types) more frequently used for cytogenetic studies (blood lymphocytes, skin fibroblasts, chorionic villi etc.) can also provide for supporting hypotheses suggesting chromosomal mosaicism to be a possible genetic mechanism underlying different human diseases [4,5,7,10,11,14,17,18,25,26]. Moreover, related studies have shed light on the understanding of the nature of some monogenic diseases that are observed in males despite the lethality (i.e. Rett syndrome) [27]. Regardless these achievements chromosomal mosaicism is still poorly described phenomenon. The latter is acknowledged to be related to technical problems encountered during attempts to detect chromosomal mosaicism [4,5]. Addressing the technical side of molecular cytogenetic analysis of somatic genome variations, one can come to the rueful conclusion that current achievements in the field are exiguously appreciated leading, thereby, to slowing down the somatic genome variation research. Looking at recent advances in interphase cytogenetics, it is to note that powerful methodological basis for high-resolution surveys of chromosomal mosaicism does exist [28]. Fortunately, examples of such studies are present in the available literature [6,11-13,15,17,28]. In this context, it is to mention the development of a molecular cytogenetic technique (interphase chromosome-specific multicolor banding) providing for visualization of the whole interphase chromosome in a cell [6,13,29], exemplified by Figure 1. Thus, researchers of somatic genome variation have to pay attention to these molecular cytogenetic developments.

**Figure 1:**
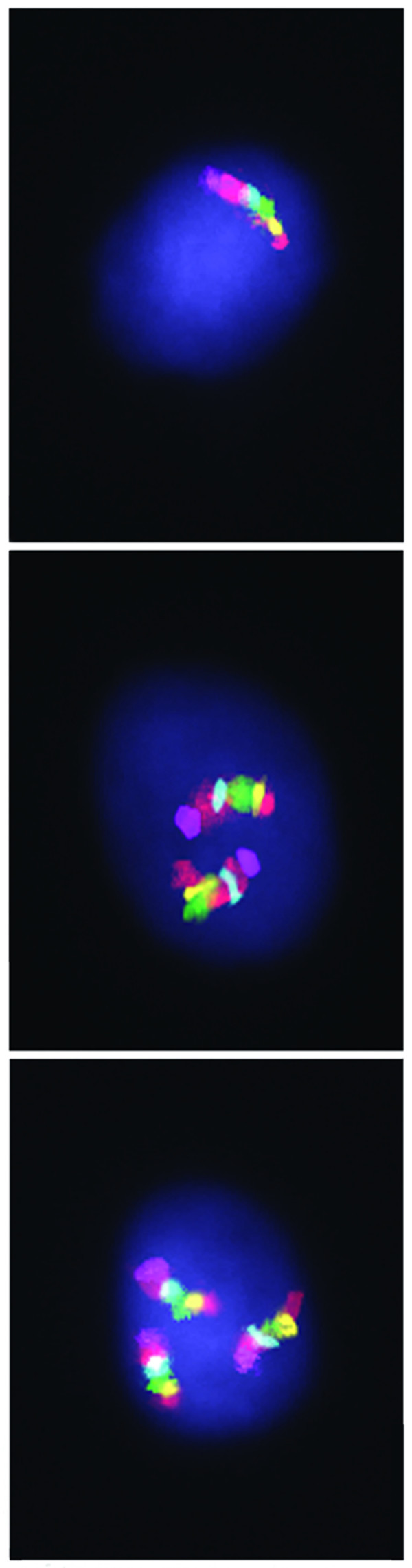
Aneuploidy in the fetal human brain. Interphase chromosome-specific multicolor banding (ICS-MCB) allowing bar-coding painting of the whole chromosome 9 in its integrity; from left to right: monosomy, disomy (normal chromosomal complement) and trisomy (partially reproduced from Yurov et al. [13], an open-access article distributed under the terms of the Creative Commons Attribution License).

**Table 2:** The load of chromosomal mosaicism to human prenatal mortality and postnatal morbidity

Condition/disease	Description	References
Spontaneous abortions	~25% of all spontaneous abortions (~50% of spontaneous abortions with chromosome abnormalities) exhibit chromosomal mosaicism	[17]

Chromosomal syndromes	3–18% (depending on chromosome)	[4,5,7]

Mental retardation and/or multiple congenital malformation	~3.5% in institutionalized children	Vorsanova & Yurov, unpublished observations

Autism	16% in children with autism (~10% X chromosome aneuploidy in male children)	[11]

Schizophrenia	Mosaic aneuploidy of chromosomes 1, 18 and X in cells of the schizophrenia brain; mosaic X chromosome aneuploidy in blood lymphocytes	[7,8,12]

Autoimmune diseases	Monosomy of chromosome X in systemic sclerosis (6.2% of cells) and autoimmune thyroid disease (4.3% of cells)	[10]

Alzheimer disease	over 10% in brain cells; increase of aneuploidy of chromosome 21 in mitotic cells (skin fibroblasts or blood lymphocytes)	[25,26]

Meiotic aneuploidy	Chromosomal mosaicism confined to fetal ovarian tissues has potential to result into meiotic aneuploidy in conceptions	[15]

Since chromosomal mosaicism is more likely to manifest as aneuploidy, it appears important to delineate the way aneuploidy occurs during the ontogeny or "aneuploidzation pathway" (Figure 2). Current data suggest aneuploidization to represent a process that accompanies human development. Being a devastative condition, aneuploidy causes prenatal death and/or chromosomal syndromes associated with severe developmental delays hardly compatible with life [4]. The human central nervous system development depicts that aneuploidy should be cleared, unless a pathogenic condition is produced [13]. Therefore, an "antianeuploidization" process (see legend to Figure 2) does exist in human, which is required for a human conception to develop into a newborn and, subsequently, to develop through the postnatal period of life. However, "antianeuploidization" seems to slowdown during human aging probably associated with aging or tumorigenesis. The latter is supported by current concepts in cancer and aging research [14,30]. The aneuploidization pathway seems, therefore, to be a kind of universal cascade of processes that leads to human disease, depending on the performance of the opposition processes, which we have arbitrarily called "antianeuploidization". Contrariwise, a balance between aneuploidization and "antianeuploidization" provides human organism to develop normally unless the "antianeuploidzation" will slow down (Figure 2). We suggest that aneuploidization of a tissue should be the key process to produce the dysfunction. Being confined to the specific cell population, it probably causes tumorigenesis, whereas the whole tissue affected by aneuploidy should degenerate. This is partially supported by the data on brain diseases [4,12,25]. Notwithstanding, such attracting hypotheses concerning aneuploidization, that assume chromosomal mosaicism to be associated with human diseases, are to be tested.

**Figure 2:**
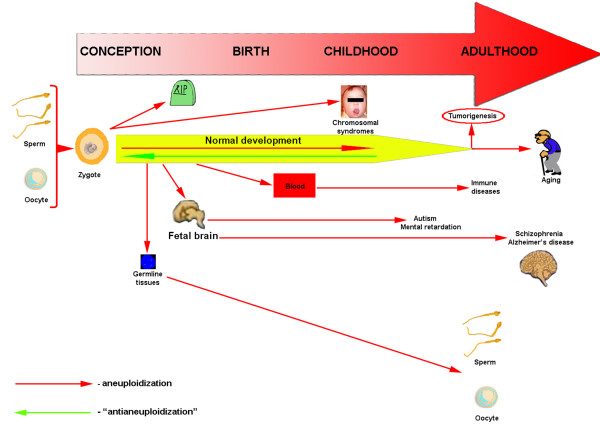
Current concepts in biology of chromosomal mosaicism: somatic-germline aneuploidization pathway. Normal prenatal and postnatal development is hypothesized to be a matter of balance between two progressive processes: aneuploidization and "antianeuploidization" (the latter is arbitrarily covered by such term because it is still not completely clear what processes underlie the clearance of aneuploid cells in humans). Germline aneuploidzation results into prenatal death of aneuploid embryos or into chromosomal syndromes in newborns. Aneuploidization is observed in fetal germline tissues and in the fetal brain. This, if not cleared, has the potential to produce tissue-specific chromosomal mosaicism that can underlie the pathogenesis of brain diseases either in childhood or in adulthood. It also can be the reason of germline aneuploidization (mentioned earlier). Aneuploidization in adulthood (in some cases, in childhood) is suggested to be a key process of tumorigenesis and aging. This probably originates from the age-/environment-dependant inhibition of "antianeuploidization" processes.

The report that has inspired this communication addresses basic side of chromosome mosaicism research. However, *Molecular Cytogenetics *has published a series of original researches, which have paid attention to practical side of chromosomal mosaicism [31-36]. These have demonstrated that chromosomal mosaicism is an appreciable phenomenon frequently encountered in small supernumerary marker chromosomes (sSMC) research [31-33,35]. Furthermore, it provided evidences that mosaic structural chromosome rearrangements are likely to occur more frequently, than previously recognized [4,5,34,36]. In the light of studying sSMC, it should be additionally mentioned that chromosomal mosaicism could be cryptic [37,38] and dynamic [39]. The former is referred to as occurrence of more complex mosaics than revealed after karyotyping [37]. The latter is the occurrence of new genetic imbalances from an already abnormal cell or mosaicism resulting from behavioral peculiarities of a rearranged chromosome [39]. These two types of chromosomal mosaicism require the application of high-resolution molecular cytogenetic techniques, i.e. subcenM-FISH or multicolor banding (MCB) [37-39]. This takes us back to the technical side of chromosome mosaicism detection and forces to conclude again that studying chromosomal mosaicism without taking into account new molecular cytogenetic techniques is almost useless. Here, it is to mention high-resolution genome screening approaches based on array-CGH. Such molecular cytogenetic techniques are extremely powerful for delineation of chromosomal breakpoints, identification of new microdeletion syndromes, and uncovering genomic variations in health and disease [7]. Related possibilities have made array-CGH-based techniques almost the most popular ones in current medical genetics. However, related approaches are poorly inapplicable (or even completely inapplicable) for uncovering low-level, cryptic and dynamic mosaicism. Therefore, genome screens by array-CGH miss cases of chromosomal mosaicism. This point should to be considered by researchers who plan to study this type of intercellular (somatic) genomic variations, as well.

Finishing our overview of chromosomal mosaicism in the light of the latest biomedical achievements, it is to highlight several points: (i) intercellular variations manifesting as chromosomal mosiacism are likely to be involved in the genetic diversity; (ii) significant proportion of human pathogenic conditions are associated with chromosomal mosaicism; (iii) chromosomal mosaicism is still underappreciated biomedical phenomenon that requires additional evaluations ; (iv) current molecular cytogenetics possesses sufficiently powerful tools for uncovering the role of chromosomal mosaicism. Together, it suggests future biomedical research to involve studies of chromosomal mosaicism, which have the potential to give us new insights into pathobiology of human diseases and to help our understanding of the intercellular genomic variations.

## Competing interests

The authors declare that they have no competing interests.

## Authors' contributions

IYI wrote the manuscript and SGV and YBY contributed significant editorial input and original ideas.
